# The epidemiology of HIV infection among female sex workers in Nairobi, Kenya: A structural determinants and life-course perspective

**DOI:** 10.1371/journal.pgph.0001529

**Published:** 2024-01-08

**Authors:** Tara S. Beattie, Wendy Adhiambo, Rhoda Kabuti, Alicja Beksinska, Pauline Ngurukiri, Hellen Babu, Mary Kung’u, Chrispo Nyamweya, Anne Mahero, Maisha Fiti Study -Champions, Erastus Irungu, Peter Muthoga, Janet Seeley, Joshua Kimani, Helen A. Weiss, Rupert Kaul

**Affiliations:** 1 Department of Global Health and Development, London School of Hygiene and Tropical Medicine, London, United Kingdom; 2 Partners for Health and Development in Africa, Nairobi, Kenya; 3 Africa Health Research Institute, KwaZulu-Natal, South Africa; 4 UNITID, University of Nairobi, Nairobi, Kenya; 5 Department of Infectious Disease Epidemiology and International Health, MRC International Statistics and Epidemiology Group, London School of Hygiene and Tropical Medicine, London, United Kingdom; 6 Department of Immunology, University of Toronto, Ontario, Canada; The University of Texas Health Science Center at Houston School of Public Health - San Antonio Campus, UNITED STATES

## Abstract

High HIV prevalence among female sex workers (FSWs) is heavily influenced by structural determinants (e.g. criminalisation of sex work; violence) and significant life events (e.g. orphanhood, teenage pregnancy). This study aims to understand the epidemiology of HIV among FSWs in Nairobi, Kenya using a structural determinants and life-course perspective. Baseline cross-sectional survey data were collected June–December 2019 for the Maisha Fiti study with 1003 FSWs (aged 18–45 years). Odds ratios and 95% confidence intervals were estimated using multivariable logistic regression with a hierarchical modelling approach. HIV prevalence was 28.0%, and increased with age (<25 years 5.7%, 25–34 years 19.0%, ≥35 years 40.6%). In adjusted analyses, HIV seroprevalence was associated with childhood and adolescence including violence from militia or soldiers (AOR = 1.60; 95%CI:1.00–2.53), young age at sexual debut (≤15 years old vs. ≥18 years AOR = 0.57; 95%CI:0.39–0.84) and teenage pregnancy (AOR = 1.37; 95%CI:1.00–1.88). For adulthood the factors included lower SES score (lowest vs. highest tertile AOR = 0.63; 95%CI:0.40–0.98); reduced housing insecurity (AOR = 0.52; 95%CI:0.54–0.79); lower alcohol/drug use score (AOR = 0.44; 95%CI:0.31–0.61); and a longer duration of selling sex (0–5 years vs. ≥11 years AOR = 2.35; 95%CI:1.44–3.82). Among HIV-negative FSWs, prevalence of HIV risk factors was high (recent hunger 32.3%; internalised 67.7% and experienced 66.0% sex work stigma; recent police arrest 30.1%; recent physical or sexual violence 65.6%, condomless last sex intimate partner 71.1%; harmful alcohol or substance use 49.1%). Only 24.6% of HIV-negative FSWs reported taking PrEP. Taken together, adverse events in childhood and adolescence were associated with increased odds of living with HIV, and were more strongly associated with HIV serostatus than adulthood structural or behavioural risk factors. HIV-negative FSWs remain at high risk of HIV acquisition. This study highlights the importance of addressing adverse events throughout the life course to reduce HIV risk, and the need to continue multi-level HIV prevention and treatment efforts.

## Introduction

In 2021, it was estimated that 38.4 million adults and children were living with HIV globally, with 20.6 million of these living in East and Southern Africa [1,2]. In 2021, almost half (44.6%) of new HIV infections globally were in people living in those regions [2]. The majority of countries in sub-Saharan Africa (SSA) have seen a decline in both HIV prevalence and incidence among the general population during the last two decades, although this progress may be slowing [1,3]. Female sex workers (FSWs) are among the most at-risk populations for HIV globally, with an estimated prevalence of 10.4% (95% CI 9·5–11·5) globally, and 33.3% (95% CI 29·2–37·6) in East and Southern Africa [4]. In 2019, FSWs had 30 times the risk of acquiring HIV compared with the general female population [5]. In Kenya, from where the data for this paper are drawn, HIV prevalence in 2021 was estimated as 29.3% among FSWs compared with 4.3% in the general population [6]. Unlike the general population, HIV prevalence among FSWs over the past decade has remained stable across most settings globally [4,7,8], with FSWs not equally benefitting from efforts to increase HIV service coverage [9]. FSWs who have sought HIV prevention and treatment services have reported violence, police harassment and discrimination from healthcare providers, all of which can deter them from accessing the care they require [10].

The high prevalence of HIV among FSWs in SSA is heavily influenced by structural determinants [10–13], defined as factors that are external to the individual and operate outside the locus of their control [14]. For HIV, known structural determinants among FSWs have been conceptualised by Shannon et al. [14] as operating at the (i) macrostructural (e.g. social, economic and health policies, and laws governing sex work, migration, stigma, cultural norms on gender and sexuality), (ii) community organisation of sex work (e.g. sex work collectivisation and empowerment) and (iii) work environment levels (e.g. venue based characteristics, managerial practices, local policing, coverage and access of condoms, HIV/STI testing and anti-retroviral therapy (ART)). These structural determinants can dynamically interact and influence inter-personal (e.g. partner-level/dyad-level risks and protections, such as condom negotiation, sexual networks and patterning) and individual factors of sex workers, clients and their non-paying intimate partners including (i) behavioural (e.g. drug use, duration in sex work), (ii) biological (e.g. age, sex, race, HIV characteristics, STI co-infection) and (iii) host genotypic factors (e.g. host immunity). Together these work to shape HIV acquisition and transmission dynamics and epidemic trajectories at the individual and population levels [14]. In particular, the criminalisation of sex work in 116 countries globally not only undermines HIV prevention, treatment, care and support efforts [12,13,15–18] but also means that FSWs have little recourse to report violence or abuse, including police violence and extortion [7,19,20]. However few studies among FSWs in SSA have included a structural determinants framework when examining associations with HIV [21]. In addition, life course theory—which takes into account significant events over someone’s entire life, including during childhood and adolescence, and which explicitly states the temporal ordering of exposure variables and their inter-relationships with the outcome measure–has rarely been applied to HIV studies among FSWs in SSA [22]. Instead, studies have tended to focus on individual and inter-personal behavioural and biological risks such as host immunity, condom use, hormonal contraceptive use and client volume. This paper helps address this evidence-gap by examining HIV risk through a structural determinants and a life-course lens.

In 2010, the Kenya National AIDS and STI Control Programme (NASCOP) developed a set of National Guidelines in response to the Kenya National HIV Strategic Plan (KNASP III), which identified FSWs as a key at-risk group for HIV [8]. Kenya’s current National HIV Programme for FSWs is a combination prevention approach that includes not only bio-medical and behavioural, but also structural interventions, and follows global guidance for programming for key populations [23]. This includes the prioritization of FSWs for PrEP and ART programming [3], although retention on daily oral PrEP has been challenging. Encouragingly, estimates suggest that HIV incidence has been decreasing among some groups of FSWs in Kenya [6,24–26]. In Nairobi county, HIV prevalence in new FSW clinic enrolees peaked at 81% in 1986, but has been consistently below 50% since 1997 [24]. Approximately 39,600 women are estimated to sell sex, and HIV prevalence is estimated as 29.5% [27,28]. A 2015 HIV incidence study by McKinnon and colleagues [29] found that HIV acquisition was associated with shorter baseline duration of sex work, minimum charge/sex act, *Neisseria gonorrhoea* infection, sex with casual clients during menses, DepoProvera use, and unprotected regular partner contacts. As this was an incidence study using clinic-level data, structural determinants and childhood and adolescent experiences were not examined.

*Maisha Fiti* is a longitudinal, mixed-methods study which aims to examine the impact of violence experiences and harmful alcohol use on systemic and genital inflammation. In 2019, we conducted a baseline behavioural-biological survey among a representative sample of 1003 FSWs in Nairobi county. We collected structural, inter-personal and individual-level data including on childhood, adolescent, and adult events. The aim of the current study is to use the baseline cross-sectional survey data to understand the epidemiology of HIV among FSWs in Nairobi, Kenya, using a structural determinants and life-course perspective. Specifically, we examine (i) structural, inter-personal (sex work) and individual risk factors across the life-course associated with HIV serostatus, and (ii) the prevalence of HIV risk behaviours among HIV-negative participants.

## Materials and methods

### Study design

The Maisha Fiti study was designed in consultation with the FSW community in Nairobi, and peer educators and staff working at seven Sex Work Outreach Programme (SWOP) clinics. The data analysed in this paper are from the baseline behavioural-biological surveys, conducted June to December 2019.

### Subjects and recruitment

SWOP clinics are health clinics specifically for FSWs across Nairobi county. Seven clinics serve approximately 29,000 (73%) of an estimated 39,000 sex workers, providing an accessible and free comprehensive package of HIV prevention and treatment services. Additional programmes provide services to other FSWs. Eligible women were aged 18–45 years, had attended one of the SWOP clinics in Nairobi in the past 12 months, were not pregnant or breast-feeding and did not have an underlying chronic illness (other than HIV) which could affect their immunology (diabetes, rheumatoid arthritis, asthma, TB infection, recent chirotherapy (to treat cervical cell abnormalities)). Women aged <25 were over-sampled to enable stratified analyses by age-group.

Sample size calculations have been described previously [30]. Eligibility of the 29,000 FSWs enrolled at a SWOP clinic was initially assessed using anonymized SWOP patient data. Of 10,409 eligible women, 1200 were randomly selected with probability weighted to clinic client volume, so that the sample was self-weighting. Selected women were contacted by telephone, told about the study, and those interested in participating were scheduled for a screening appointment at the dedicated study clinic in downtown Nairobi. At the screening visit, eligible women received detailed information about the study both verbally and through a written participant information leaflet. Consenting women undertook a pregnancy test, and those who were not pregnant or breast-feeding were enrolled in the study and completed a behavioural-biological survey.

### Behavioural-biological survey

All interviews were conducted in Swahili or English by a trained research assistant or clinician. The behavioural questionnaire took approximately 1–1.5 hours to complete and contained sections on: childhood experiences, socio-demographic factors, sexual and reproductive health factors, violence, stigma, sex work and behavioural characteristics. Biological samples comprised: a urine to test for pregnancy (exclusion criteria) and for *Neisseria gonorrhoeae* (NG) and *Chlamydia trachomatis* (CT) using GeneXpert Assay. HIV status was screened by rapid HIV tests, with positive tests confirmed using HIV DNA Genexpert. Blood samples were collected to confirm new HIV diagnoses and to assess *Treponema pallidum* (syphilis) infection using the plasma reagin assay. Genital swabs were used to test for *Trichomonas vaginalis* (TV; OSOM Trichomonas Rapid Test; SEKISUI Diagnostics, LLC) and *Bacterial vaginosis* (BV; Gram’s stain and Nugent scoring). Given that the questionnaire contained sensitive questions, the study team underwent an intensive three-week training prior to the study start, which included pilot testing and refining the questionnaire and developing clear referral pathways for women who required additional support. Participants who reported recent violence, mental health problems or suicidal behaviours were referred to a trained counsellor employed as part of the study team. Women reporting ongoing violence experiences were also linked to the violence response team at the SWOP clinics. All women who tested positive for HIV were counselled and referred for HIV care at their chosen SWOP clinic. Women who tested positive for STIs were offered treatment free of charge. Participants were reimbursed for their time. Questionnaires were completed on paper, and double-entered using CS-PRO.

### Measurements

The main outcome variable of interest was HIV serostatus (defined using the laboratory tests described above).

**Fig 1:**
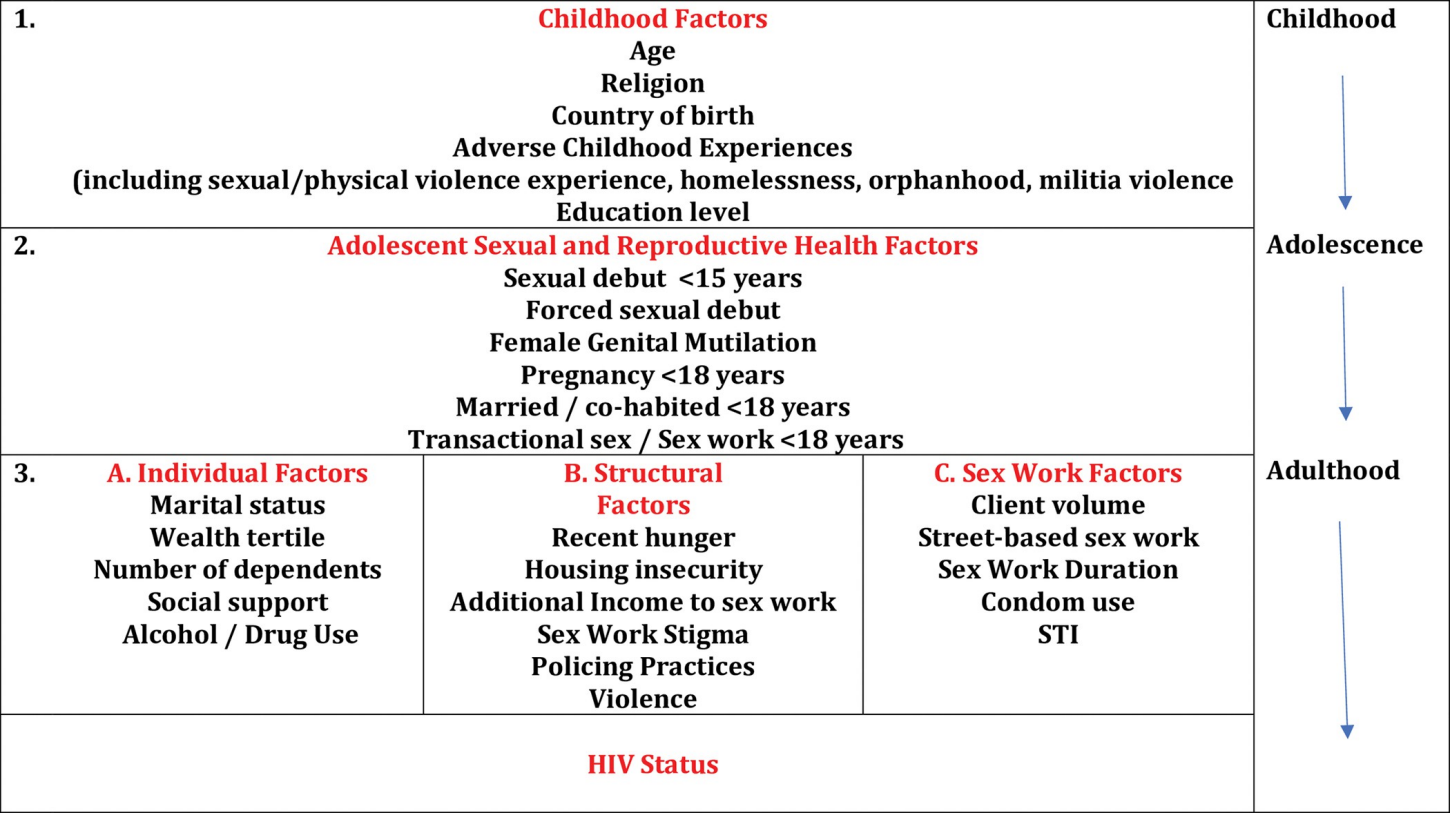
Conceptual hierarchical framework of risk factors across the life course for current HIV status among FSWs in Nairobi, Kenya.

To examine risk factors across the life-course associated with HIV status (objective 1), we developed a conceptual framework, drawing on current theories about risk factors for HIV among FSWs, using a structural determinants and life-course perspective (Fig 1). Thus we diagrammatically ordered classes of variables across the life course, prior to statistical analysis. We conceptualised risk factors as operating across the life course (childhood, adolescence and adulthood) and at a variety of hierarchical levels (structural, inter-personal (sex work) and individual) [14,31–33]. Variables could be both ‘childhood’ *and* ‘structural’ e.g. education level; forced sexual debut, as well as ‘adulthood’ *and* ‘structural’ e.g. sex work stigma, police arrest. Exposure variables are shown in Fig 1, with further details on variable measurement provided in Table 1.

**Table 1:** Definition of exposure variables used in the Maisha Fiti study with FSWs in Nairobi, Kenya.

Variable	Tool / Question	Category
*Childhood Factors*		
ACEs	WHO Adverse Childhood Experiences International Questionnaire (ACE-IQ) [34] Due to the length of the questionnaire, three questions from the WHO ACE-IQ tool were not included (one question on bullying from peers and two questions on emotional and physical neglect from parents/guardians were excluded). An additional question about street homelessness in childhood was incorporated as it was considered relevant for this population.	Individually and Ordered categorical variable (each ACE scores one point): <4 (low), 5–8 (moderate), 9–12 (high) [35]
*Adolescent Factors*		
Forced sexual debut	Would you say you wanted to have sex that first time, or was it against your will?	I wanted to have sex vs. I was tricked into having sex or I was pressured into having sex vs. I was physically forced to have sex
Female Genital Mutilation	Have you ever had a surgical procedure used for modifying the vagina or restoration of the hymen, including female genital circumcision, incision with insertion of substance into the lesion?	No vs. Yes
*Adulthood Factors*		
Wealth Tertiles	14 household asset questions used in the Kenyan Demographic Health Surveys [36]	Principle component analysis (PCA) used to compute household wealth tertiles: Low, Medium, High
Number of dependents	Not including yourself, how many people living in your household are dependent on your income? How many people outside your household re dependent on your income?	0–1, 2–3, ≥4 dependents
Social support	Do you have someone who you can talk to about your problems?	Yes/Sometimes vs. No
Recent hunger	Thinking now about the past 7 days, have you or anyone in your family skipped a meal because there was not enough food?	No vs. Yes
Housing insecurity	In the past one year, how many times have you moved house?	0–1 vs. ≥2
Sex Work Stigma—Internalised	“I have lost respect or standing in the community because I sell sex”, “I think less of myself because I sell sex” and /or “I have felt ashamed because I sell sex”. (Ever (but not in the past 12 months) and past 12 months)	No vs. Yes
Sex Work Stigma—Experienced	“People have talked badly about me because I sell sex”, “I have been verbally insulted, harassed or threatened because I sell sex”, and/or “I have felt excluded from or rejected by my family because I sell sex”. (Ever (but not in the past 12 months) and past 12 months)	No vs. Yes
Sex Work Stigma—Anticipated	“I have avoided seeking health services because I am worried someone may learn I sell sex”. (Ever (but not in the past 12 months) and past 12 months)	No vs. Yes
Police arrest	Have you been arrested (i) ever and/or (ii) in the past 6 months because you are a sex worker?	No vs. Yes
Violence	WHO Violence Against Women Questionnaire [37] adapted to ask about violence from intimate partners and non-intimate partners ever and in the past 6 months. Physical or sexual violence defined as answering yes to any of the eight questions on physical of sexual violence	No vs. Yes
Harmful alcohol use	WHO ASSIST (Alcohol, Smoking and Substance Involvement Screening Test) tool [38]	Low risk 0–10; moderate risk ≥11; high risk >27
Harmful substance use	WHO ASSIST (Alcohol, Smoking and Substance Involvement Screening Test) tool [38]	Low risk 0–4; moderate risk >4; high risk >27
STI	Testing positive for “any STI” was defined as a positive diagnostic test for *T*. *pallidum*, *N*. *gonorrhoeae*, *C*. *trachomatis* or *T*. *vaginalis*.	No vs. Yes

**Fig 2:**
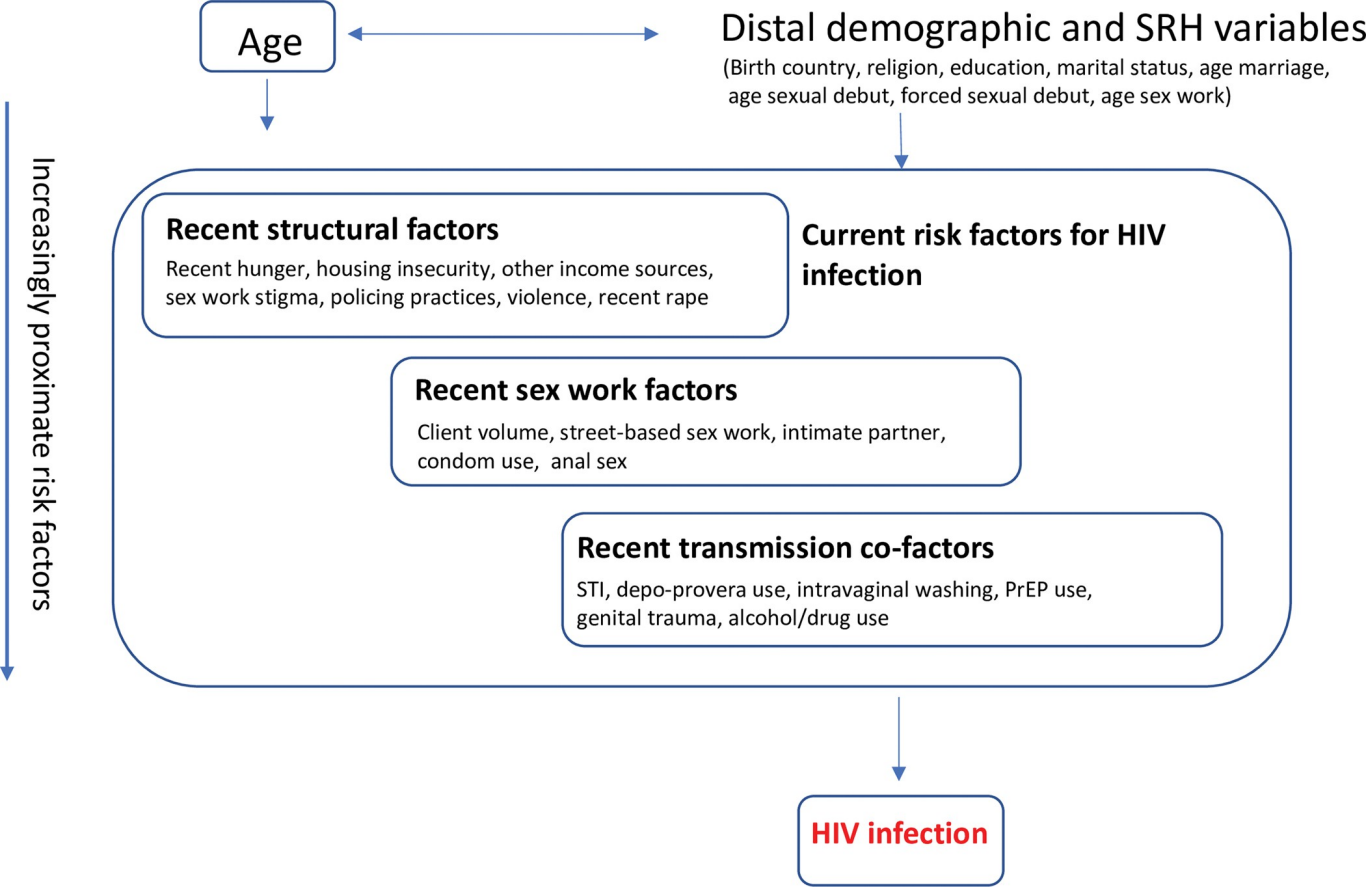
Hierarchical conceptual framework to examine the potential effect and relationships between age and HIV infection among HIV negative FSWs in Nairobi, Kenya.

To examine the prevalence of HIV risk behaviours among HIV-negative participants (objective 2), we developed a conceptual framework based on the literature, and drawing on life-course and structural determinants theory, and grouped exposure variables into three domains (Fig 2). Current risk factors for HIV infection were diagrammatically ordered as operating at the structural, sex work and individual (transmission co-factors) levels. Five intravaginal washing practices were ascertained (yes/no) using standardised questions [39].

### Statistical analysis

Data were double entered and statistical analyses were conducted in STATA version 16.1. (Stata Inc., College Station, TX, USA). Data were weighted to allow for over-sampling of participants aged <25 years old. To adjust for clustering by clinic, SWOP clinic was included as a fixed effect in all models. Descriptive statistics and the Wald test were used to describe the study cohort and HIV prevalence overall. Associations with HIV serostatus (objective 1) were estimated using odds ratios (OR) and 95%CI, with p-values obtained using a joint hypothesis test via the adjusted Wald test (to allow for sampling weights). Informed by the structural determinants and life-course theories, we categorised variables into three hierarchical levels (i) childhood factors (ii) adolescent sexual and reproductive and health factors and (iii) adulthood risk factors. We examined associations of these with HIV status using logistic regression. Age was included in all models as an a-priori confounder of other exposures of interest. We built models starting with variables earliest in the life-course and worked forward time-wise, so that we first estimated the direct effects for childhood and adolescent variables on HIV serostatus, independent of any mediating effect through more recent adulthood variables [40]. We then examined the direct effects of more recent adulthood variables on the outcome, after adjusting for upstream confounders. Thus, following the conceptual framework (Fig 1), we built multivariable models for the outcome (HIV serostatus) as follows: In model 1, the overall associations of HIV with level 1 variables (childhood factors) were examined and a core group adjusted for each other were retained if associated with HIV status (p-value <0.1). In model 2, level 2 variables (adolescent sexual and reproductive health factors) were examined adjusted for the core level 1 variables, age and clinic, and retained if independently associated with HIV status (p-value <0.1). Similarly, in models 3a-3c, level 3a-3c variables (adulthood individual, structural, and sex work (inter-personal) variables, respectively) were adjusted for the core level 1 and 2 variables and retained if independently associated with HIV status (p-value <0.1). Missing data were reported if >5% of observations were missing.

To examine the prevalence of risk behaviours among HIV-negative participants (objective 2), we conducted weighted descriptive analyses informed by our conceptual framework (Fig 2), overall and stratified by age. We stratified by age as we were a-priori interested in understanding if risk behaviours differed by age. P-values were obtained using a joint hypothesis test via the adjusted Wald test.

### Ethics statement

All participants provided written formal consent. The Maisha Fiti study was approved by the Kenyatta National Hospital Ethics and Research Committee (KNH ERC P778/11/2018), the London School of Hygiene and Tropical Medicine (LSHTM) Ethics Committee (Approval number: 16229) and the University of Toronto ethics committee (Approval number: 37046).

## Results

### Participant characteristics and HIV and STI status, overall and by age

Of 1200 women randomly sampled from 7 SWOP clinic sites in Nairobi, 1039 (86.6%) were eligible for the study, of whom 1003 (96.5%) were enrolled. Participant characteristics are detailed in Table 2. Almost all participants were born in Kenya (98.7%) and most were Protestant (54.4%) or Catholic (36.8%). Overall, 28.4% had an ACE score of 0–4 (low), 54.3% had an ACE score of 5–8 (moderate) and 17.3% had an ACE score of 9–12 (high), with little difference by age group (p-value = 0.767). Around two-thirds of participants (69.2%) had primary education or less, with increasing education levels among younger age groups (p-value <0.001) (Table 2). Most participants (81.3%) had previously been married, and lived alone or with children (83.0%); 59.8% said that they currently had an intimate partner.

**Table 2:** Participant characteristics overall and by age.

		Total		Age Group			
		N (1003)	%	<25 years % (n = 212)	25–34 years % (n = 353)	≥35 Years % (n = 438)	P- value**
***Socio-demographic characteristics***							
*Country of birth*	Kenya Other	989 14	98.7 1.3	98.1 1.9	99.2 0.9	98.4 1.6	0.469
*Religion*	Catholic Protestant Muslim Other/ none	374 535 46 48	36.8 54.4 4.6 4.2	41.5 44.3 4.7 9.4	36.8 53.5 4.8 4.8	35.6 54.4 4.3 2.5	0.014
*Socio-Economic Score*	Lower Middle Upper	334 334 333	32.6 33.5 34.0	40.1 32.6 27.4	33.7 32.6 33.7	29.8 34.5 34.0	0.011
*ACE Score*	0–4 (low) 5–8 (moderate) 9–12 (high)	282 548 173	28.4 54.3 17.3	25.5 57.6 17.0	27.5 54.4 18.1	29.9 53.4 16.7	0.767
*Education*	Did not complete primary Completed primary but not secondary Completed secondary or higher	169 525 309	16.9 52.3 30.8	9.0 52.4 38.7	15.9 51.8 32.3	21.5 52.7 25.8	<0.001
*Ever been married*	No Yes	2125 788	18.7 81.3	44.3 55.7	21.0 79.0	10.7 89.3	<0.001
*Current living status*	Lives alone or with children Lives with friends or parents Lives with male partner	832 103 67	83.0 10.3 6.7	62.7 31.6 5.7	85.3 6.8 7.9	91.1 2.8 6.2	<0.001
*Current Intimate partner*	No Yes	392 610	40.2 59.8	30.3 69.7	40.8 59.2	42.2 57.8	0.035
***HIV and STIs***							
HIV	*Positive*	257	28.0	5.7	19.0	40.6	<0.001
*Syphilis*	*Positive*	20	2.1	0.9	1.4	3.0	0.122
*Gonorrhoea*	*Positive*	27	2.6	3.3	2.3	2.6	0.751
*Chlamydia*	*Positive*	67	6.7	15.2	7.4	2.1	<0.001
*Any bacterial STI* *	*Yes*	136	13.6	20.3	13.0	10.7	0.011
*Trichomonas*	*Positive*	31	3.0	3.8	2.3	3.6	0.448
*BV*	*Positive*	199	20.0	19.4	19.7	20.4	0.928

All percentages are weighted to allow for over-sampling of <25 year olds.

*Syphilis, gonorrhoea and/or chlamydia.

**χ^2^ Test or Test for trend for ordered categorical variables.

HIV prevalence was 28.0%, and increased with age from 5.7% among those aged <25 years to 40.6% among those aged ≥35 years (p-value <0.001) (Table 2). Prevalence of bacterial STIs was relatively low: syphilis (2.1%), gonorrhoea (2.6%), chlamydia (7.6%), and was higher in younger compared with older women (p-value = 0.011). BV prevalence was 20.0%, and trichomonas prevalence was 3.0%, with no evidence of a difference by age group (p-value = 0.928; p-value = 0.448, respectively).

### Associations with HIV status

We first examined risk factors associated with HIV serostatus, using a structural determinants and life-course perspective (objective 1) (Fig 1, Table 3). In adjusted analyses there was strong evidence that HIV serostatus was independently associated with increasing age (25–34 years vs. <25 years; AOR = 3.51; 95%CI:1.86–6.63; ≥35 years vs. <25 years: AOR = 9.77; 95%CI:5.23–18.27), and experiencing violence from soldiers in childhood (AOR = 1.60; 95%CI:1.00–2.53) (Model 1). There was weak evidence that low education level (completed secondary school or higher vs. did not complete primary school (AOR = 0.64; 95%CI:0.40–1.02)), was also associated with increased odds of HIV infection (Model 1). In model 2, HIV serostatus was independently associated with the following adolescent sexual and reproductive health risk factors: early age of sexual debut (age of sexual debut ≥18 years vs. ≤15 years AOR = 0.57; 95%CI:0.39–0.84) and early age at first pregnancy (<18 years of age vs ≥18: AOR = 1.37; 95%CI:1.00–1.88). When we examined associations between HIV serostatus and more recent adulthood risk factors, after adjusting for factors in models 1 and 2, in Model 3a, HIV serostatus was associated with being in the lowest compared with the highest wealth tertile (AOR = 0.63; 95%CI:0.40–0.98; test for trend; p = 0.041) and having low compared with medium/high levels of alcohol or drug use (AOR = 0.44: 95%CI:0.31–0.61). In model 3b, having fewer compared with ≥2 house moves in the past year (AOR = 0.52; 95%CI: 0.34–0.79) was strongly associated with HIV serostatus. In model 3c, we found evidence that increasing duration in sex work was associated with HIV serostatus (test for trend: p = 0.001) with HIV serostatus strongly associated with ≥11 years of sex work compared with selling sex for 0–5 years (AOR = 2.12; 95%CI: 1.31–3.44).

**Table 3:** Multivariable logistic regression–associations with HIV status.

			All N 1003	All %*	HIV negative % (n = 746)	Living with HIV % (n = 257)	Odds Ratio Adjusted for age (95% CI)	Additionally adjusted for other variables in each level (95% CI)	P-value **
*Model 1* ***	*Age*	*<25* *25–34* *≥35*	212 353 438	11.7 39.4 48.9	94.3 81.0 59.4	5.7 19.0 40.6	Ref 3.90 (2.07–7.35) 11.41 (6.22–20.94)	Ref 3.51 (1.86–6.63) 9.77 (5.23–18.27)	<0.001
	*Religion*	*Catholic* *Protestant* *Muslim* *Other/ none*	374 534 46 46	36.9 54.4 4.6 4.1	73.6 70.0 75.6 78.3	26.4 30.0 24.4 21.7	Ref 1.12 (0.82–1.53) 0.89 (0.43–1.84) 1.02 (0.442.36)		
	*Country of birth*	*Kenya* *Other*	989 14	98.7 1.3	72.1 66.6	27.9 33.4	Ref 1.23 (0.40–3.76)		
	*Education*	*Did not complete primary* *Completed primary but not secondary* *Completed secondary or higher*	169 525 309	17.8 52.3 29.9	68.3 69.6 78.3	31.7 30.4 21.7	Ref 1.09 (0.74–1.60) (0.48–1.14)	Ref 0.99 (0.66–1.49) 0.64 (0.40–1.02)	0.033
	*Orphanhood in childhood*	*No* *Yes*	595 408	58.6 41.4	74.3 68.8	25.7 31.3	Ref 1.34 (1.00–1.80)	Ref 1.29 (0.95–1.74)	0.091
	*Lived on the Streets in childhood*	*No* *Yes*	878 125	88.0 12.0	72.4 68.8	27.6 31.2	Ref 1.45 (0.93–2.26)		
	*Sexual or Physical Violence in childhood*	*No* *Yes*	234 769	23.0 77.0	72.5 71.8	27.5 28.2	Ref 1.04 (0.73–1.48)		
	*Beaten up by soldiers in childhood*	*No* *Yes*	899 104	89.5 10.5	73.1 62.4	26.9 37.6	Ref 1.59 (1.00–2.51)	Ref 1.60 (1.00–2.53)	0.047
	*Adverse Childhood Experience (ACE) Score*	*0–4 (low)* *5–8 (moderate)* *9–12 (high)*	282 548 173	28.4 54.3 17.3	66.8 76.2 67.4	33.2 23.8 32.6	Ref 0.64 (0.46–0.89) 1.03 (0.67–1.59)		
*Model 2* ***	*Age at Sexual Debut (years)*	*≤15 years* *16–17 years* *≥18 years*	369 312 315	37.3 30.0 32.7	65.7 72.3 78.3	34.3 27.7 21.7	Ref 0.77 (0.54–1.09) 0.50 (0.35–0.72)	Ref 0.81 (0.56–1.16) 0.57 (0.39–0.84)	0.005
	*Consensual sexual debut*	*Yes* *No*	695 304	68.8 31.2	74.2 67.1	25.8 32.9	Ref 1.25 (0.92–1.69)		
	*Female genital mutilation*	*No* *Yes*	943 60	93.7 6.3	72.5 64.6	27.5 35.4	Ref 1.15 (0.66–1.99)		
	*Eldest child conceived when <18 years*	*No* *Yes*	517 486	52.4 47.7	75.0 68.7	25.1 31.3	Ref 1.62 (1.21–2.18)	Ref 1.37 (1.00–1.88)	0.052
	*Married or co-habited <18 years*	*No* *Yes*	759 244	75.8 24.3	73.1 68.5	26.9 31.5	Ref 1.31 (0.94–1.83)		
	*Sex work debut <18 years*	*≤17* *≤18*	176 815	16.6 83.4	72.5 72.0	27.5 28.0	Ref 0.89 (0.60–1.32)		
*Model 3A* ***	*Ever Married*	*No* *Yes*	216 787	18.8 81.2	82.2 69.6	17.8 30.4	Ref 1.37 (0.89–2.09)		
	*Tertiles SES score*	*Lower* *Middle* *Upper*	*334* *334* *333*	*32*.*6* *33*.*5* *34*.*0*	71.7 69.4 75.3	28.3 30.6 24.7	Ref 1.03 (0.72–1.47) 0.73 (0.51–1.05)	Ref 0.91 (0.62–1.34) 0.63 (0.40–0.98)	0.041
	*Number of people dependent on her income*	*0–1* *2–3* *≥4*	178 475 350	15.4 48.6 36.0	76.8 72.0 69.9	23.3 28.0 30.1	Ref 0.64–1.60) 1.02 (0.64–1.64)		
	*Has someone can talk to*	*No* *Yes*	278 725	27.5 72.5	72.4 71.8	27.6 28.2	Ref 0.96 (0.69–1.34)		
	*Alcohol use or drug use score*	*None / Low* *Medium / High*	553 450	56.4 43.6	65.1 80.9	34.9 19.1	Ref 0.51 (0.38–0.70)	Ref 0.44 (0.31–0.61)	<0.001
*Model 3B* ***	*Hunger in the past 7 days*	*No* *Yes*	670 331	66.1 33.9	73.7 68.5	26.3 31.5	Ref 1.17 (0.86–1.59)		
	*Housing insecurity past year*	*0-1 house moves* *≥2 house moves*	804 199	*80*.*6* *19*.*5*	69.8 81.1	30.2 18.9	Ref 0.57 (0.38–0.86)	Ref 0.52 (0.34–0.79)	0.002
	*Additional income to sex work*	*No* *Yes*	*571* *432*	*56*.*4* *43*.*7*	70.9 73.4	29.1 26.6	Ref 0.79 (0.59–1.06)		
	*Internalised sex work stigma ever*	*No* *Yes*	*272* *721*	*27*.*3* *72*.*7*	72.3 71.7	27.7 28.3	Ref 1.07 (0.76–1.49)		
	*Experienced sex work stigma ever*	*No* *Yes*	*295* *694*	*29*.*5* *70*.*6*	73.5 71.2	26.5 28.8	Ref 1.17 (0.85–1.61)		
	*Anticipated sex work stigma ever*	*No* *Yes*	*852* *142*	*86*.*2* *13*.*8*	72.0 71.0	28.0 29.0	Ref 1.17 (0.75–1.81)		
	*Police arrest ever*	*No* *Yes*	437 563	41.6 58.4	77.3 68.3	22.7 31.7	Ref 1.38 (1.02–1.87)	Ref 1.30 (0.94, 1.79)	0.113
	*Police incarceration ever*	*No* *Yes*	*848* *149*	*84*.*2* *15*.*8*	73.0 67.0	27.0 33.0	Ref 1.31 (0.88–1.94)		
	*Police sexual abuse ever*	*No* *Yes*	867 133	86.3 13.7	72.3 70.6	27.8 29.4	Ref 0.97 (0.64–1.48)		
	*Intimate partner physical or sexual violence (ever)*	*No* *Yes*	443 560	43.8 56.3	70.0 73.5	30.0 26.5	Ref 0.90 (0.67–1.20)		
	*Non-Partner physical or sexual violence (ever)*	*No* *Yes*	315 688	30.8 69.2	71.9 72.0	28.1 28.0	Ref 0.94 (0.69–1.29)		
	*Gang rape (ever)*	*No* *Yes*	912 91	90.5 9.5	72.7 65.5	27.3 34.5	Ref 1.26 (0.78–2.05)		
*Model 3C* ***	*Client volume last week*	*<5* *5–9* *≥10*	607 250 137	60.9 24.8 14.2	69.2 78.5 73.5	30.9 21.5 26.5	Ref 0.65 (0.45–0.94) 0.79 (0.51–1.22)	Ref 0.67 (0.45–0.98) 0.72 (0.46–1.13)	0.069
	*Street based sex work*	*No* *Yes*	705 294	70.0 30.0	72.8 70.3	27.3 29.7	Ref 1.20 (0.87–1.64)		
	*Duration of selling sex work*	*0–5 years* *6–10 years* *≥11years*	349 262 387	29.1 27.6 43.3	86.9 78.0 58.1	13.1 22.0 41.9	Ref 1.24 (0.77–2.00) 2.36 (1.49–3.73)	Ref 1.30 (0.45–2.12) 2.12 (1.31–3.44)	0.001
	*Condom use last sex (any partner)*	*No* *Yes*	236 765	22.8 77.2	76.3 70.8	23.8 29.2	Ref 1.22 (0.85–1.76)		
	Bacterial STI prevalence****	*None* *≥One*	891 112	89.8 10.2	71.0 80.9	29.0 19.1	Ref 0.70 (0.41–1.20)		

*Percentages weighted to allow for oversampling of <25 year olds.

**Adjusted wald test, except for the following ordered categorical variables where a test for trend is reported: age; education level; age at sexual debut; tertiles SES score; duration of selling sex.

***Model 1: *Childhood factors*. Adjusted for age and clinic, and for other variables in model 1 associated with HIV status in univariate analyses (p<0.1). Model 2: *Adolescent sexual and reproductive health risk factors*. Adjusted for age and clinic, final Model 1 variables and for variables in model 2 associated with HIV status in univariate analyses (p<0.1). Model 3A: *Adulthood individual risk factors*. Adjusted for age and clinic, final Model 1 and 2 variables and for variables in model 3A associated with HIV status in univariate analyses (p<0.1). Model 3B: *Adulthood structural risk factors*. Adjusted for age and clinic, final Model 1 and 2 variables and for variables in model 3B associated with HIV status in univariate analyses (p<0.1). Model 3C: *Adulthood sex work risk factors*. Adjusted for age and clinic, final Model 1 and 2 variables and for variables in model 3C associated with HIV status in univariate analyses (p<0.1).

****Tested positive for syphilis, chlamydia and/or gonorrhoea infection.

### HIV risk factors among HIV negative women

We next described the prevalence of current HIV risk behaviours among HIV-negative participants, using a structural determinants and life-course approach (objective 2) (Fig 2, Table 4). When we considered adulthood structural factors, one third (32.3%) of HIV negative participants reported food insecurity in the past week and one fifth (21.9%) reported housing insecurity in the past year. One third had more than 4 people dependent on their income, and just over half (55.5%) had no additional income to sex work. Both the number of dependents and recent food insecurity increased with increasing age (p-value = 0.001; p-value = 0.027, respectively). Two thirds of women reported internalised (67.7%) and experienced (66.0%) sex work stigma in the past 12 months, 30.0% reported recent police arrest and 6.9% reported having had sex with the police to avoid arrest in the past 6 months. Nearly two-thirds of HIV-negative participants (65.6%) reported physical or sexual violence in the past 6 months, with physical or sexual violence most common among women aged 25–34 years (69.9%) (p-value = 0.067). A substantial minority (6.3%) reported being raped in the past 7 days.

**Table 4:** HIV risk factors among HIV negative female sex workers in Nairobi, Kenya.

		All %* (n = 746)	<25 years % (n = 200)	25–34 years % (n = 286)	≥35 years% (n = 260)	*P value (Adjusted Wald Test)* **
***Recent structural factors***						
*Self or family skipped a meal in past 7 days as no food*	Yes	32.3	25.1	29.1	38.5	0.027
*Moved house 2+ times past year*	Yes	21.9	23.5	21.7	21.5	0.578
*Number of dependents on her income*	0–1 2–3 4+	16.4 48.6 35.0	38.0 36.0 26.0	12.6 54.2 33.2	12.3 47.3 40.4	<0.001
*Additional income to sex work*	Yes	44.5	40.5	42.7	48.1	0.243
*Internalised sex work stigma past 12 months*	Yes	67.7	65.5	71.0	65.0	0.492
*Experienced sex work stigma past 12 months*	Yes	66.0	61.0	69.6	63.9	0.133
*Anticipated sex work stigma past 12 months*	Yes	9.1	11.0	9.1	8.5	0.354
*Police arrest past 6 months*	Yes	30.1	23.5	35.0	27.3	0.036
*Police incarceration past 6 months*	Yes	6.4	2.0	7.0	7.3	0.012
*Had sex with police to avoid arrest past 6 months*	Yes	6.9	5.5	6.6	7.7	0.423
*Intimate partner physical or sexual violence past 6 months*	Yes	33.8	31.0	35.0	33.5	0.399
*Non-Partner physical or sexual violence past 6 months*	Yes	54.4	50.5	57.0	53.1	0.256
*Any perpetrator physical or sexual violence past 6 months*	Yes	65.6	59.5	69.9	63.1	0.067
*Gang rape past 6 months*	Yes	2.3	1.5	1.8	3.1	0.460
*Rape in the past 7 days*	Yes	6.3	3.5	7.3	6.2	0.095
***Recent sex work factors***						
*Clients volume previous week*	<5 5+	58.5 41.6	61.9 38.1	55.2 44.8	60.7 39.3	0.311
*Solicits clients from the street*	Yes	29.3	23.9	33.6	26.5	0.088
*Current non-paying Intimate Partner*	Yes	63.2	71.5	60.5	63.1	0.013
*Condom use last vaginal sex with client*	Yes	83.2	80.1	84.6	82.8	0.240
*Condom use last vaginal sex with Intimate Partner (n = 480)*	Yes	28.9	22.4	26.6	34.2	0.078
*Anal sex with client last 6 months*	*Yes*	4.1	3.6	4.9	3.5	0.678
***Recent HIV transmission co-factors***						
*Syphilis*	Positive	1.8	0.5	0.7	3.5	0.175
*Chlamydia*	Positive	7.4	16.1	8.4	3.1	<0.001
*Gonorrhoea*	Positive	2.6	3.5	2.8	1.9	0.392
*Trichomonas*	Positive	2.6	3.5	1.8	3.1	0.405
*Any STI* ***	Positive	13.5	20.5	13.3	11.1	0.005
*Bacterial Vaginosis*	Positive	17.1	19.1	18.7	14.6	0.445
*Depo-provera use*	Yes	20.0	27.5	21.7	15.4	0.009
*Used soap*, *antiseptic*, *lemon*, *cloth to clean or inserted drying substance into vagina*, *past 30 days*	Yes	37.9	48.0	36.0	36.2	0.003
*Currently taking PREP*	Yes	24.6	22.0	24.9	25.3	0.378
*Harmful alcohol or substance use*	Moderate /high	49.1	57.0	54.9	39.6	0.022

*Percentages weighted to allow for oversampling of <25 year olds.

**Adjusted for clinic.

*** Syphilis, Chlamydia, Gonorrhoea or Trichomonas.

In terms of adulthood sex work factors, 41.6% reported having had 5 or more clients in the previous week and 29.3% reported soliciting clients from the streets, with street-based sex work most likely among women aged 25–34 years old (p-value = 0.088). Reported condom use at last sex with a client was 83.2%, but condom use with intimate partners was lower (28.9%). Anal sex with clients was reported by a small minority (4.1%). When we examined HIV transmission co-factors, 13.5% of HIV negative women had at least one STI and 17.1% had BV. The prevalence of STIs was highest among the <25 year old participants (p-value = 0.005). One fifth (20.0%) of participants reported current depo-provera use, with the highest prevalence among <25 year olds (27.5%) (p-value = 0.009). Using substances to clean or dry the vagina was common, with 37.9% of women reporting having used soap, antiseptic, lemon, cloth to clean or inserted drying substance into vagina in the past 30 days. Younger women were more likely to report intravaginal washing than older women (48.0% among <25 year olds vs 36.2% among ≥35 years olds) (p-value = 0.003). One-quarter (24.6%) reported currently taking PrEP, with little difference by age group (p-value = 0.378). Almost half of participants (49.1%) had an alcohol or substance use problem, and prevalence was highest among those aged <25 years (p-value = 0.022).

## Discussion

In this study among Kenyan FSWs, we found that key events in childhood and adolescence, such as violence from soldiers, early age at first sex and early age at first pregnancy, were associated with HIV serostatus, with weak evidence that not completing secondary school may also be a risk factor. These events were more closely related to HIV risk than more recent structural risk factors such as food insecurity, sex work stigma, police arrest, violence and gang-rape. The inverse association with alcohol and drug use problems has previously been reported by this team and is likely due to the intensive counselling that FSWs living with HIV receive by the SWOP clinic teams, due to the negative impacts of alcohol and drug use on anti-retroviral compliance and efficacy [41].

A review by Shannon et al. of 87 FSW studies which explicitly examined structural determinants of HIV noted that few such studies have been published in SSA [21]; our study adds to this literature. Education is one structural factor included in several SSA FSW studies; similar to here, studies with FSWs in Zimbabwe, Cameroon, South Africa, Nigeria, Mombasa, and Uganda, found that secondary school education or higher may be protective, although no associations between education level and HIV were found among FSWs in Lesotho, The Gambia or Rwanda [42–50]. Historical gender based violence (physical or sexual) was examined in our study, as well as with FSWs in Zimbabwe and Rwanda, with positive associations found between forced sex and HIV in Rwanda only [43,48]. Sex-work related stigma was examined in our study, and in Cameroon and Lesotho, with positive associations with enacted sex work stigma seen in Lesotho only [42,44]. At the work environment level, physical or sexual violence by clients, police, managers or pimps was examined in our study, as well as in Cameroon, The Gambia and Zimbabwe, with no associations with HIV seen in any of these settings [42,43,45]. However, police arrest or incarceration (ever) was positively associated with HIV in Cameroon and Rwanda (but not in our study) [42,51]. In addition, to our knowledge, ours is the first study from SSA to examine HIV risk among FSWs from a life-course perspective. Previous studies from South Africa and Malawi among young adults from the general population have reported associations between a higher ACE or childhood trauma score and HIV risk behaviours [52,53]. We did not find associations between HIV and overall ACE score [54], although there was evidence that individual items (violence from soldiers or militia during childhood) were associated with HIV serostatus. Taken together, these findings highlight the importance of supporting adolescents and young women who experience key life events such as war, young age at sexual debut and teenage pregnancy to help reduce their risk of future HIV infection.

We also found that HIV negative sex workers reported high levels of exposure to recent structural HIV risk factors (food and housing insecurity, having two or more other people dependent on their income, internalised and experienced sex work stigma, recent police arrest, recent physical or sexual violence experience). We also found high levels of exposure to sex work risk factors (condom use particularly with regular partners) and HIV transmission co-factors (testing positive for an STI and/or bacterial vaginosis; PrEP use; harmful alcohol or drug use). Taken together, these findings suggest that many HIV negative FSWs in this setting remain at risk of HIV acquisition. The study with young FSWs in Zimbabwe reported similar concerns [43].

Since 2003, the Kenyan government, supported by overseas donors have implemented targeted HIV prevention interventions both for the general population, and for key populations including FSWs [17]. From 2008, these have included the widespread implementation of HIV testing and free ART services, the provision of FSW-specific clinics and outreach, and some structural interventions, including FSW-led community mobilisation and violence response interventions [26]. Similar to other settings, the HIV prevalence among the general population has fallen dramatically in Kenya over the past 25 years from 10.5% in 1995–1996 to 4.0% in 2021, with a 53% reduction in new HIV infections between 2010 and 2021 [55,56]. In addition, the prevalence among clients of FSWs is also estimated to have fallen across SSA from 10% prior to 2001 to 3% from 2011 onwards [57], although data is limited for Kenya. Contrary to many other FSW settings globally where HIV prevalence has remained stable [4,7], the HIV prevalence among FSWs in Kenya and in Nairobi has declined over the past two decades [24–26,58]. This is likely due to sustained prevention efforts by the Government of Kenya, international donors such as the US President’s Emergency fund for AIDS Relief (PEPFAR) and the Global Fund to Fight AIDS, Tuberculosis and Malaria (GFATM). The population-level scale-up and roll-out of ART for people living with HIV, and the work of specialised sex work services and programmes implemented by more than 100 partners across Kenya will also have been important [3,8,17]. In addition, the heterogeneity in host HIV susceptibility may have contributed to this decline [59]. However, our findings, together with the literature suggest that we cannot become complacent; in order to prevent steep increases in new HIV infections with increasing age, multi-level FSW HIV intervention efforts need to continue. In addition, our findings suggest that interventions which address harmful alcohol and drug use as part of HIV prevention planning, as well as the decriminalisation of sex work, are also needed [12,17].

A key limitation of our study was the cross-sectional study design, which limits our ability to ascertain the direction of causality. This is particularly important when examining recent determinants of HIV risk, where HIV infection is likely to have preceded factors such as recent housing or food insecurity and current STI prevalence. Cohort studies are needed to determine causality but these can be challenging to conduct with hidden and hard-to-reach populations such as FSWs. In addition, large sample sizes and/or a long follow-up will be needed in most settings; while the *Maisha Fiti* study is a longitudinal study, the sample size is too small to be able to conduct HIV incidence analyses. Although our sample was random, it was drawn from FSWs registered at SWOP clinics across Nairobi county; we may have missed the most vulnerable FSWs who are not registered at a SWOP clinic, such as young or new FSWs. This may have led to an under-estimate of key HIV risk factors such as STI prevalence, violence and harmful alcohol/drug use. Many of our questions were sensitive, including those on ACEs, violence and mental health and this could have led to an underestimate of key exposure variables. The study team underwent three weeks of intensive training and were supported by weekly de-brief meetings throughout data collection. In addition, the study team included 10 FSWs employed as outreach workers at the SWOP clinics and as beauticians and receptionists at the study clinic. The SWOP clinics were established in 2010 and have built trust with the FSW community, especially *vis a vis* confidentiality. Together these should have helped reduce interviewer and reporting bias. Key strengths of this study was the large sample size and the random selection of FSWs from across Nairobi county. The comprehensive behavioural-biological survey data meant we were able to examine several variables across the life-course in our analyses.

### Conclusions

This study adds to newly emerging literature on the importance of childhood and adolescent experiences as well as structural factors on shaping the HIV risk environment for high risk groups such as FSWs. We also demonstrate that despite national and county-level targeted HIV intervention programming for key populations and considerable declines in HIV prevalence over time, many HIV negative FSWs in this setting remain at high risk of HIV infection. Together this calls for the continuation of targeted multi-level FSW programming, which addresses structural as well as behavioural and biological determinants of risk. ‘Upstream’ HIV prevention programmes which can identify and specifically work with adolescents and young adults who have experienced adverse life events such as violence from soldiers or militia, early sexual debut and/or teen pregnancy are also needed.
